# Multilocus sequence typing of *Treponema pallidum* in male patients with genital ulcers in a public sexually transmitted infections clinic: a new allele and almost complete macrolide resistance^☆^

**DOI:** 10.1016/j.abd.2024.10.008

**Published:** 2025-04-28

**Authors:** Leonardo Souza Esteves, Vera Mileide Trivellato Grassi, Liliane Trivellato Grassi, Maria Rita Castilhos Nicola, Marcia Susana Nunes Silva, Maria Lucia Rosa Rossetti, Mauro Cunha Ramos

**Affiliations:** a Laboratory of Molecular Biology Applied to Microbacteria, Fundação Oswaldo Cruz, Rio de Janeiro, RJ, Brazil; b Program in Cellular and Molecular Biology Applied to Health Sciences, Universidade Luterana do Brasil, Canoas, RS, Brazil; c Biomedical Engineering, Universidade Luterana do Brasil, Canoas, RS, Brazil; d Sanitary Dermatology Outpatient Unit, Secretaria Estadual de Saúde, Porto Alegre, RS, Brazil; e DNTech - Development of New Technologies LTDA, Agência Estadual de Pesquisa, Desenvolvimento e Inovação do Rio Grande do Sul / Financiadora de Estudos e Projetos, Porto Alegre, RS, Brazil; f Postgraduate Program, Faculdade de Biologia Celular e Molecular Aplicada à Saúde, Universidade Luterana do Brasil, Canoas, RS, Brazil

Dear Editor,

Syphilis is a sexually transmitted infection of polymorphic evolution caused by the *Treponema Pallidum* subspecies *pallidum* (TP). Mucocutaneous, neurological and cardiovascular systems are the most affected and mother-to-child transmission can occur at any stage of pregnancy.^1^ The World Health Organization (WHO) estimated 6.3 million yearly cases in the world. In Brazil, syphilis increased from 33.9 cases in 2015 to 74.2 cases per 100,000 inhabitants in 2019.^2^

TP is non-cultivable with standard culture methods. In clinical practice, the diagnosis is presumptive using serological tests. Different Polymerase Chain Reactions (PCR) techniques have been used for diagnosis^3^ and DNA sequencing is increasingly used to study genetic diversity, dynamics of transmission, virulence, and patterns of resistance.^4^ Genotyping by Multilocus Sequence Typing (MLST) on the chromosomal loci TP0136, TP0548, and TP0705 allows better discrimination of TP strains and permits the creation of an epidemiological analysis database (https://pubmlst.org/organisms/treponema-pallidum). Different loci, distinct alleles, and their combination define the allelic profile and the Sequence Type (ST). The analysis of the 23SrRNA gene can complement the identification of mutations (A2058G or A2059G) that are related to resistance to macrolides.^5^

The objective of our study is detecting and genotype TP in by MLST Genital Ulcers Presumptive of Syphilis (GUPS).

We analyzed exudate GUPS samples obtained from male patients aged 18 years and over, seen at a public STI clinic in Porto Alegre, Brazil, from July 2019 to March 2019. Sample collection: dry cotton swab. DNA extraction: PureLink® Genomic Kit (Invitrogen®, Thermo Fisher Scientific). Detection: PCR amplification of 260 bp of the tpp47 gene with DNA using the *primers* KO3 (5'-GAAGTTTGTCCCAGTTGCTGCTTT-3') and KO4 (5'-CAGAGCCATCAGCCCTTTTCA-3'). Amplification: *Taq platinum* DNA polymerase (Invitrogem, Fisher Scientific, USA) and Thermocycler PTC96 (Bioer Technology, China). PCR product analysis: electrophoresis in agarose gel (0.05% ethidium-bromide). MLST analysis: sequencing chromosomal loci (TP0136, TP0548, and TP0705) as described previously by Vrbová et al.,^4^ Evaluation of A2058G and A2059G mutations in the 23S rRNA gene: nested PCR. Sequencing analysis: Bioedit software (Tom Hall, USA). Genotyping analysis: scheme including TP0136, TP0548, and TP0705 loci,^4^ using the public database platform for molecular typing and microbial genome diversity (PubMLST, available at https://pubmlst.org/organisms/treponema-pallidum). Resistance-related sequencing analysis: sequence annotation corresponding to positions 2058 and 2059 in rRNA gene of *Escherichia coli* (accession nº V0033, GenBank). Positive and negative controls were used in each round. Ethical approval: School of Public Health, state of Rio Grande do Sul (nº 3,232,889).

Forty-three participants were recruited. Ages ranged from 19 to 66 years. All of them were residents of the metropolitan area of Porto Alegre. In 32 (74%) of the 43 DNA samples analyzed, we detected TP specific sequences. In 30 (94%) of the 32 at least one locus of the MLST scheme was successfully sequenced. We successfully sequenced the same proportion 23SrRNA gene. We obtained quality sequencing of TP0705 in 22 samples, as well as for TP0136 and TP0548. The combination of successfully sequenced locus among samples varied. We identified three allele variants for TP0136, two for TP0548, and three for TP0705. We identified a new allele for TP0705 in the only sample characterized as genotypically susceptible to macrolides (designated allele 11) which differed from allele 3 at loci position 270 (C270 T). Three other samples presented heterozygous peaks (two peaks), at positions 154 for TP0136, 14 for TP0548, and 265 for TP0705 (Fig. 1). The results of TP typing, the identified alleles, the genotypic resistance profile, and the clonal complex are presented in Table 1.

**Figure 1:**
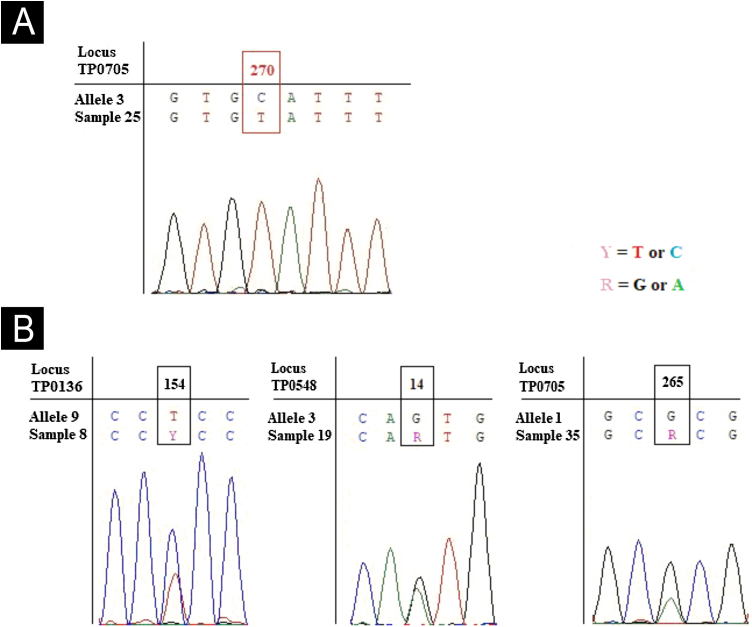
**New allele** for TP0705 and three heterozygous peaks for TP0136, TP0548 and TP0705 loci. (A) Chromatogram of sample 25 shown the new allele variant called here of 11, characterized by an SNV at position 270 (C→T) using the allele 3 (most similar sequence) as reference. (B) Chromatograms of three samples showed heterozygous peak for TP0136 locus at position 154 (C and T), TP0548 at position 14 (G and A) and TP0705 at position 265 (G and A). The alleles used as reference are the most similar sequence observed in PubMLST database.

**Table 1:** Genotyping of *T. pallidum* by Multi-Locus Sequence Typing (MLST) of studied samples.

Sample ID	TP0136	TP0548	TP0705	23S rRNA^a^	MLST^b^	ST^c^	Clonal Complex
1	1	3	1	R	1.3.1	1	SS14-like
7	nd	nd	1	R	x.x.1	‒	SS14-like^d^
8	9^f^	7		R	9.7.x	‒	Nichols-like^d^
9	1	3	‒	R	1.3.x	‒	SS14-like^d^
10	1	nd	1	R	1.x.1	‒	SS14-like^d^
11	1	3	1	R	1.3.1	1	SS14-like
12	1	3	nd	R	1.3.x		SS14-like^d^
13	nd	3	nd	R	x.3.x		SS14-like^d^
14	nd	nd	nd	R	‒	‒	nd
15	nd	nd	nd	R	‒	‒	nd
16	28	7	3	R	28.7.3	‒	Nichols-like
17	nd	nd	1	R	x.x.1	‒	SS14-like^d^
18	1	3	1	R	1.3.1	1	SS14-like
19	1	3^f^	nd	R	1.3.x	‒	SS14-like^d^
20	1	3	nd	R	1.3.x		SS14-like^d^
21	nd	7	3	R	x.7.3	‒	Nichols-like^d^
22	9	7	3	R	9.7.3	26	Nichols-like
23	nd	nd	3	nd	x.x.3	‒	nd
25	nd	nd	11^e^	S	x.x.11	‒	nd
27	1	3	nd	R	1.3.x	‒	SS14-like^d^
28	1	3	1	R	1.3.1	1	SS14-like
29	28	7	3	R	28.7.3	‒	Nichols-like
30	1	3	1	R	1.3.1	1	SS14-like
31	nd	nd	1	R	x.x.1	‒	SS14-like^d^
32	1	3	1	R	1.3.1	1	SS14-like
34	1	nd	1	Nd	1.x.1	‒	SS14-like^d^
35	1	nd	1^f^	R	1.x.1	‒	SS14-like^d^
36	28	7	3	R	28.7.3	‒	Nichols-like
38	1	3	nd	R	1.3.x	‒	SS14-like^d^
39	1	3	1	R	1.3.1	1	SS14-like
40	1	3	1	R	1.3.1	1	SS14-like
42	nd	3	1	R	x.3.1	‒	SS14-like^d^

nd, Not determined.

a 23S rRNA gene encoding resistance to macrolide antibiotics: S, Sensitive; R, Resistant with mutation A2058G.

b Allelic profiles based on TP0136, TP0548 and TP0705 loci sequences.

c ST, Sequence Type, according to the PubMLST database for *Treponema pallidum subsp. Pallidum*.

d Clonal complex classified by approximation.

e New allele.

f Alleles with heterozygous peak in one position of the sequence.

We obtained Sequence Types (ST), which are attributed to fully characterized haplotypes (three MLST loci), for 11 samples and the most frequent (8/11;72,7%) was the profile 1.3.1 (ST 1). Profile 28.7.3 has not yet attributed an ST in PubMLST platform. We classified samples that had at least one locus successfully sequenced in a clonal complex by approximation. This classification was based on the number of sequenced isolates containing the allele(s) stored in the PubMLST database (Table 2). We identified two clonal complexes of strains: 6 (20%) of 30 isolates were classified as Nichols-like and 22 (73%) as SS14-like. It was not feasible to assign a clonal complex to the two samples that only had the TP0705 locus successfully sequenced. One sample was characterized as allele 3, while the other exhibited a newly identified allele designated as allele TP0705-22 (Fig. 1).^5^ Regarding resistance-related mutations, out of the 30 samples that were adequately characterized, only one did not have the A2058G mutation in the 23S rRNA gene and therefore classified as susceptible.

**Table 2:** Clonal complex of incomplete genotypic profiles based on number of samples present in PubMLST database.

Profile searched in the database	n of samples SS14-like	n of samples Nichols-like	n of samples without CC assignment	CC considered in this study
1.3.x	463	0	1	SS14-like
1.x.1	639	0	10	SS14-like
9.7.x	0	38	0	Nichols-like
x.3.1	477	0	4	SS14-like
x.3.x	482	0	6	SS14-like
x.7.3	0	41	3	Nichols-like
x.x.1	683	0	20	SS14-like
x.x.3	22	86	7	nd

CC, Clonal Complex.

The TP DNA was detected in approximately two-thirds of the samples. They belonged to the SS14-like or Nichols-like clonal complexes. A new allele was identified in the two samples not classified in a clonal complex (x.x.3 and x.x.11).^5^ The haplotype containing this allele had only the TP0705 locus characterized. This locus can share identical alleles among strains from SS14 and Nichols-clades, which makes it difficult to assign a clonal complex.

All but one of our DNA-positive samples presented the A2058G mutation in the 23S rRNA gene, which provides resistance to the macrolide class of antibiotics.^6^ Our findings corroborate those of Grillová et al.^7^ and Giacani et al.,^8^ which demonstrated a high and increasing proportion of this mutation. We identified the same clonal complexes in 20% of the samples of this study (Clonal Complex SS14 or the Nichols-like). We found three MLST fully characterized profiles of TP (1.3.1; 9.7.3; 28.7.3).

The negative samples to TP DNA can be explained: 1) Some patients did not have syphilis; 2) Microorganisms in the lesions reduced over time; and 3) The previous use of topical and systemic antibiotics. The small sample size prevented the study associations of different genotypes with demographics, sexual practices, or geographic origin of participants since the SARS-CoV2 epidemic hindered the enrollment of participants. The development of studies with larger sample sizes will be able to provide additional information that is crucial for the control of syphilis.

## Financial support

The *Coordenação de Aperfeiçoamento de Pessoal de Nível Superior* of the Ministry of Education (CAPES) provided a scholarship for doctoral studies of V.M.T. Grassi through funding CODE 001. The Brazilian Ministry of Health funded the study through a Letter of Agreement with the Pan-American Health Organization (PAHO) number SCON00400/2019.

## Authors’ contributions

Leonardo Souza Esteves: Responsible for the conception and design, carried out bioinformatic sequencing analysis and genotyping, provided comments and edits and approved the final draft prior to submission.

Vera Mileide Trivellato Grassi: Responsible for the conception and design, provided comments and edits and approved the final draft prior to submission.

Liliane Trivellato Grassi: Carried out reference organizing, provided comments and edits and approved the final draft prior to submission.

Maria Rita Castilhos Nicola: Participated in data collection and management, provided comments and edits and approved the final draft prior to submission.

Marcia Susana Nunes Silva: Responsible for the conception and design, provided comments and edits and approved the final draft prior to submission.

Maria Lucia Rosa Rossetti: Responsible for the conception and design, provided comments and edits and approved the final draft prior to submission.

Mauro Cunha Ramos: Responsible for the conception and design, provided comments and edits and approved the final draft prior to submission.

## Conflicts of interest

None declared.
